# Application of blended care as a mechanism of action in the construction of digital therapeutics

**DOI:** 10.31744/einstein_journal/2020MD5640

**Published:** 2020-11-12

**Authors:** Antonio Valerio

**Affiliations:** 1 Universidade Federal de São Paulo Escola Paulista de Medicina São PauloSP Brazil Escola Paulista de Medicina, Universidade Federal de São Paulo, São Paulo, SP, Brazil.

**Keywords:** Telemedicine, mHealth, Therapeutics

## Abstract

The article describes the use of blended care as a mechanism of action in the construction of a therapy that uses digital means to improve and support current clinical treatments. The so-called digital therapeutics provides evidence-based therapeutic interventions for patients, who are guided by software to prevent, manage, or treat a medical disorder or disease. They are used independently or together with drugs, devices, or other therapies to optimize patient care and health outcomes. They are similar to popular wellness applications, but with one key difference: they focus on providing clinical results. Broadly speaking, they are evidence-based behavioral treatments delivered online, which can increase accessibility of health care and its efficacy. It is a combination of activities involving face-to-face care and digital care, aiming to provide follow-up of patients’ performance in their self-care. Regarding blended care, digital therapeutics acts as an extension of physical care. They are complementary, regardless of the proportion used of each, in the care process of the assisted individual. It works as a bridge between the traditional provision of face-to-face care and eHealth solutions.

## INTRODUCTION

Digital therapeutics (DTx) provide evidence-based therapeutic interventions for patients, who are guided by software to prevent, manage, or treat a disorder or medical disease. They are used independently or along with drugs, devices, or other therapies to optimize patient care and health results.^(^^1^^)^

Digital therapeutics, as it is most commonly known in Brazil, is related to technology-based products that have passed through clinical validation, such as digital systems and applications, among others, and have a direct impact on the diagnosis, prevention, monitoring, or treatment of diseases, conditions, or syndromes.^(^^2^^)^ They are similar to the popular well-being applications (apps), but with a fundamental difference: they concentrate on providing clinical results.^(^^3^^)^ They are online evidence-based behavioral treatments that can increase accessibility to health care and its efficacy.

The purpose of DTx is to offer new therapy options for overlooked medical needs to patients, providers, and health insurance companies. It can be used independently or together with other therapies. It also aims to enhance and support current medical treatments, with the possibility of reducing dependence on certain pharmaceutical products. Moreover, it promotes the integration of the medical guidelines and the best practices, to provide patient experience with the best results.

The conditions to be considered a DTx are related to the incorporation of the best practices of technology linked to design, clinical validation, usability, and data safety. DTx should be validated by regulatory agencies, such as the Food and Drug Administration (FDA) or *Conformité Européenne* (CE), to answer the questions related to risk, efficacy, and intended use. A DTx needs to be clinically validated and connected to the flow of clinical work. There should be data collection and an instantaneous feedback loop. Additionally, the procedure should be paid, and the prescription must be made by a healthcare professional.^(^^1^^)^

Regarding the procedure, DTx can be divided into three parts: category, mechanism of action, and evidence-based results. The category corresponds to addressing a specific medical condition, focusing on managing or preventing a disease, and optimizing the use of drugs, and ultimately, the treatment itself of a disease or disorder. The mechanism of action corresponds to the formula that is used to make up this therapy, as if it were the set of chemical components of a drug. Therefore, each digital therapeutic has its own composition of activities to provide the expected results. The North American company Welldoc, in Columbia, *e.g.* , is focused on users with type 2 diabetes and employs four elements: personalized guidance, online connection between patient and health team, connected health devices with risk classification, and display of the medication regimen.^(^^4^^)^

In September 2017, the PEAR Therapeutics reSET, in Boston,^(^^5^^)^ which is currently marketed by Sandoz - Novartis Group, became the first FDA approved digital therapeutics. It treats people with opioid and substance use disorders. It is important to state that digital therapeutics can be prescribed with or without pharmacotherapy, always by a healthcare professional (physician, dietitian, psychologist, among others). It is important not to confuse digital therapeutics with tele-consultation or remote home services, which are very common in startup portfolios, such as Talkspace, Doctor on Demand, TeenCounseling, etc..

**Figure 1:**
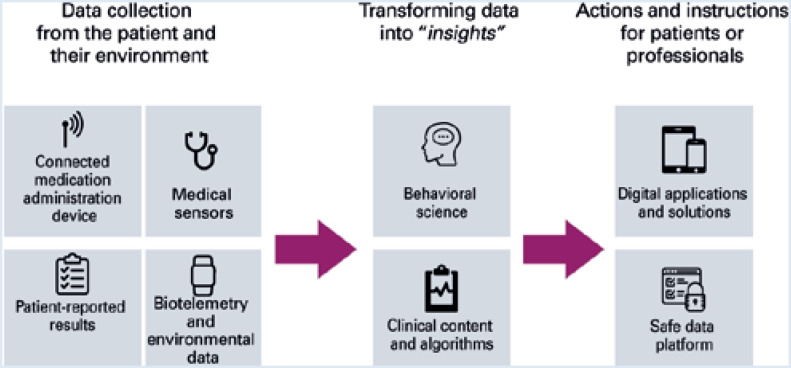
Three phases involved in digital therapeutics

The success of digital therapeutics requires three phases, as shown in figure 1 .

As mechanism of action of DTx, blended healthcare or blended care^(^^6^^)^ is proposed as an important differential for the correct treatment outcome. Blended care aims to positively modify both the patient's journey and the intervention in their experience and engagement with regard to ongoing care. It acts as a bridge between traditional face-to-face care and eHealth solutions.

In an article published at Harvard Business Review, professor Darryl Rigby cites the term “physital *”* [ *i.e* ., physical and digital], which is associated with the learning of companies that ignored the digital movement, and those that concluded the digital world would inexorably end their positions.^(^^7^^)^ The two visions were wrong, that is, more and more companies will need to have their relationship models with the client and with the market, encompassing these two environments: off-line and online. Based on this understanding, the application of a digital care method associated with physical care was proposed. Blended care is the joining of activities involving digital care, which can be related to an automatic process or not, in which electronic equipment is used for measurement and communication to provide follow-up of the elderly in support of physical care. In the blended care process, digital care acts as an extension of physical care. In this case, one complements the other, regardless of the proportion each is used in the care process of the assisted individual. For example, in a patient who is in the recovery period, digital care may correspond to 70% of interventions and 30% of a physical caregiver in person or *vice-versa* .

This type of proposal is in line with world trends in the area of telemedicine and biomedical engineering, which is the application of mHealth.^(^^8^^,^^9^^)^ It involves the use of mobile and wireless technologies, such as smartphones, smartwatches, patient telemonitoring devices, personal digital assistants, and mobile software applications (apps), to support the achievement of objectives in the field of health.^(^^10^^)^ mHealth is a subset of eHealth, and both involve the application of information and communication technologies (ICT) to support health and health-related activities.

## CONCLUSION

In general, the benefits of using digital therapeutics for users are related to providing reliable evidence-based interventions with high quality control. They can increase access to clinically proven therapies that are safe and effective, and provide care regardless of patient programming and in the privacy of their own environment - *e.g.* , at home, at work, etc.

For providers and health care systems, the benefits are found in prescribing clinical care to patients together with drugs, devices, or other therapies to optimize care and outcomes. In addition, digital therapeutics enable intelligent management of data-driven care and clinical decision-making. The purpose is to provide clinically proven therapies that offer patients effective self-management therapeutic options, as well as expanded access to evidence-based medical therapies.

In the case of health insurance companies, the benefits are in improving economic, clinical, and health outcomes for a wide variety of physical, behavioral, and mental disorders, as well as in the increased exposure of the patient population to treatments, without potentially requiring an equivalent expansion of the workforce. Furthermore, it should be possible to reduce the economic burden of medical conditions, reducing overall costs and promoting treatment options for conditions that were previously treated by traditional medicines and therapies.
