# Supplementary material for: Application of blended care as a mechanism of action in the construction of digital therapeutics
Source: Einstein (Sao Paulo). 2020 Nov 12;18:eMD5640. doi: 10.31744/einstein_journal/2020MD5640 (PMC7664824; doi:10.31744/einstein_journal/2020MD5640)
Supplement: Translation

# Translation: Aplicação do cuidado híbrido como mecanismo de ação na construção de uma terapêutica digital

**Authors:** Antonio Valerio

**Affiliations:** 2 São PauloSP Brasil Escola Paulista de Medicina, Universidade Federal de São Paulo, São Paulo, SP, Brasil.

**Keywords:** Telemedicina, mHealth, Terapêutica

## Abstract

O artigo descreve o emprego do cuidado híbrido como mecanismo de ação na construção de uma terapêutica que utiliza os meios digitais para aprimorar e apoiar os tratamentos médicos atuais. A chamada terapêutica digital fornece intervenções terapêuticas baseadas em evidências para pacientes, que são orientados por software para prevenir, gerenciar ou tratar um distúrbio ou doença médica. São usados independentemente ou em conjunto com medicamentos, dispositivos ou outras terapias para otimizar o atendimento ao paciente e os resultados de saúde. São semelhantes aos populares aplicativos de bem-estar, mas com uma diferença fundamental: concentram-se no fornecimento de resultados clínicos. Em linhas gerais, são tratamentos comportamentais baseados em evidências entregues online, que podem aumentar a acessibilidade e a eficácia dos cuidados de saúde. Trata-se da junção de atividades envolvendo o atendimento presencial e o cuidado digital, com o objetivo de prover um acompanhamento do desempenho do paciente em seu autocuidado. Sobre o cuidado híbrido, a terapêutica digital atua como extensão do atendimento físico. São complementares, não importando a proporção do uso de cada um, no processo de cuidado do indivíduo assistido. Funciona como uma ponte entre a prestação tradicional de assistência presencial e as soluções de Saúde Digital.

## INTRODUÇÃO

A terapêutica digital (DTx) fornece intervenções terapêuticas baseadas em evidências para pacientes, que são orientados por software para prevenir, gerenciar ou tratar um distúrbio ou doença médica. São usados independentemente ou em conjunto com medicamentos, dispositivos ou outras terapias, para otimizar o atendimento ao paciente e os resultados de saúde.^(^^1^^)^

A terapia digital, como é mais conhecida no Brasil, está relacionada a produtos baseados em tecnologia, que tenham passado por validação clínica, como sistemas digitais e aplicativos, entre outros, e tenham impacto direto no diagnóstico, na prevenção, no monitoramento ou no tratamento de doenças, condições ou síndromes.^(^^2^^)^São semelhantes aos populares aplicativos de bem-estar, mas com uma diferença fundamental: concentram-se no fornecimento de resultados clínicos.^(^^3^^)^São tratamentos comportamentais baseados em evidências entregues online que podem aumentar a acessibilidade e a eficácia dos cuidados de saúde.

O propósito da DTx é oferecer a pacientes, presta­dores e operadoras novas opções de terapia para neces­sidades médicas não atendidas. Pode ser utilizado in­dependentemente ou em conjunto com outras terapias. Busca também aprimorar e apoiar os tratamentos mé­dicos atuais, com a possibilidade de redução da dependência de certos produtos farmacêuticos. Promove ainda a integração das diretrizes médicas e as melhores práticas, para entregar uma experiência do paciente com melhores resultados.

As condições para ser considerada uma DTx estão relacionadas à incorporação das melhores práticas de tecnologia ligadas a design, validação clínica, usabilidade e segurança de dados. A DTx deve ser validada pelos órgãos reguladores, como o *Food and Drug Administration* (FDA) ou *Conformitè Européenne* (CE), para balizar as questões relacionadas a risco, eficá­cia e uso pretendido. Uma DTx precisa ser clinicamente validada e conectada ao fluxo de trabalho clínico. Deve existir a coleta de dados e o *loop* de *feedback* instantâneo. Ainda, o procedimento deve ser remunerado. Além disso, a prescrição deve ser reali­zada por um profissional de saúde que recomendará ou prescreverá uma DTx.^(^^1^^)^

Com relação ao procedimento, uma DTx pode ser dividida em três partes: categoria, mecanismo de ação e resultados baseados em evidências. A categoria corres­ponde a endereçamento a uma condição médica específica, foco em gerenciar ou prevenir uma doença, e em otimizar o uso de medicamentos e, por fim, o tratamento em si de uma doença ou distúrbio. O meca­nismo de ação corresponde à fórmula que é utilizada para compor essa terapia, como se fosse um conjunto de componentes químicos de um medicamento. Diante disso, cada terapia digital tem sua composição de ati­vidades para prover os resultados esperados. A em­presa norte-americana Welldoc, em Colúmbia, por exemplo, é focada em usuários com diabetes tipo 2 e utiliza quatro elementos: orientação personalizada, conexão online paciente e equipe de saúde, dispositivos de saúde conectados com classificação de risco e exibição do regime de medição.^(^^4^^)^

Em setembro de 2017, o reSET da empresa PEAR Therapeutics, em Boston,^(^^5^^)^que atualmente é comercializada pela Sandoz do Grupo Novartis, tornou-se a primeira terapia digital aprovada pelo FDA. Ela trata pessoas com transtorno de uso de opioides e de substâncias. É importante registrar que uma terapia digital pode ser prescrita com ou sem combinações com uma farmacoterapia, sempre por um profissional de saúde (médico, nutricionista, psicólogo, entre outros). É importante não confundir a terapia digital com serviços de teleconsulta ou teleatendimento, muito comum nos portfólios de *startups* como a *Talkspace, Doctor on Demand, Teen Counseling* , etc..

O sucesso de uma terapia digital requer três fases, como pode ser observado na figura 1 .

**Figura 1:** Três fases envolvendo a terapia digital

Como mecanismo de ação de uma DTx é proposto o emprego do cuidado híbrido, ( *blended healthcare* ou *blended care* ),^(^^6^^)^como diferencial importante para o correto desfecho do tratamento. O cuidado híbrido tem o objetivo de modificar positivamente tanto a jornada do paciente, quanto a intervenção em sua experiência e engajamento, com relação ao seu cuidado contínuo. Funciona como uma ponte entre a prestação tradi­cional de assistência presencial e as soluções de Saúde Digital ( *eHealth* ).

Em um artigo publicado na *Harvard Business Review* , o professor Darryl Rigby, cita o termo “fisital”, que está associado ao aprendizado de empresas que igno­raram o movimento digital, e daquelas que concluíram que o mundo digital inexoravelmente acabaria com suas posições.^(^^7^^)^As duas visões estavam equivo­cadas, ou seja, cada vez mais as empresas precisarão ter seus modelos de relacionamento com o cliente e com o mercado, contemplando esses dois ambientes: *off-line* e online. Com base nesse entendimento é que foi proposta a aplicação de um método de cuidado digital associado a um cuidado físico. O cuidado híbrido é a junção de atividades envolvendo o cuidado digital, que pode estar relacionado a um processo automático ou não, no qual se faz uso de equipamentos eletrônicos para medição e comunicação com o objetivo de prover um acompanhamento do idoso em apoio ao cuidado físico. No processo do cuidado híbrido, o cuidado digital atua como extensão do atendimento físico. Neste caso, um é complementar ao outro, não importando a proporção do uso de cada um no processo de cuidado do individuo assistido. Por exemplo, no cuidado de um paciente em recuperação, o cuidado digital pode corresponder a 70% das intervenções e a 30% de um cuidador físico presencial ou vice-versa.

Esse tipo de proposta está alinhada com as tendências mundiais na área de telemedicina e engenharia biomédica, que é a aplicação do *mHealth* .^(^^8^^,^^9^^)^Trata-se do uso de tecnologias móveis e sem fio, como *smartphones, smartwatches* , dispositivos de telemonitoramento de pacientes, assistentes pessoais digitais e aplicativos de soft­ware móveis (apps), para apoiar a realização dos objetivos da área de saúde.^(^^10^^)^O *mHealth* é um subconjunto do *eHealth* , e ambos envolvem a aplicação das tecnologias da informação e comunicação (TIC) para apoiar a saúde e as atividades relacionadas com a saúde.

## CONCLUSÃO

Em linhas gerais, os benefícios do emprego das terapias digitais aos usuários estão relacionados ao fornecimento de intervenções confiáveis baseadas em evidências, com um alto controle de qualidade. Podem aumentar o acesso às terapias clinicamente demonstradas como seguras e eficazes, além de fornecerem cuidados independentemente da programação do paciente e na pri­vacidade de seu próprio ambiente – por exemplo: na sua casa, trabalho etc.

Para os provedores e os sistemas de saúde, os benefícios se encontram na prescrição aos pacientes da assistência médica em conjunto com medicamentos, dispositivos ou outras terapias, para otimizar o atendimento e os resultados dos desfechos. Além disso, as terapias digitais permitem o gerenciamento inteligente de assistência orientada por dados e tomada de decisão clínica. O propósito é o fornecimento de terapias clinicamente comprovadas, que ofereçam aos pacientes opções terapêuticas de autogestão eficazes, assim como a expansão do acesso a terapias médicas baseadas em evidências.

No caso das operadoras de saúde, os benefícios estão na melhoria dos resultados econômicos, clínicos e de saúde para uma grande variedade de distúrbios físicos, comportamentais e mentais, e também no aumento da exposição da população de pacientes a tratamentos, sem potencialmente exigir uma expansão equivalente da força de trabalho. Ainda, deve ser possível diminuir a carga econômica das condições médicas, reduzindo os custos gerais e promovendo opções de tratamento para condições que, anteriormente, foram tratadas por medicamentos e terapias tradicionais.
